# *Gastrodiae Rhizoma* Water Extract Ameliorates Hypothalamic–Pituitary–Adrenal Axis Hyperactivity and Inflammation Induced by Chronic Unpredictable Mild Stress in Rats

**DOI:** 10.1155/2020/8374614

**Published:** 2020-06-10

**Authors:** Min Wang, Wanrui Dong, Ruiying Wang, Xudong Xu, Ying Wu, Guibo Sun, Xiaobo Sun

**Affiliations:** ^1^Institute of Medicinal Plant Development, Chinese Academy of Medical Sciences & Peking Union Medical College, Beijing 100193, China; ^2^Harbin University of Commerce, Harbin, 150076 Heilongjiang, China; ^3^Department of Traditional Chinese Medicine, Beijing Obstetrics and Gynecology Hospital, Capital Medical University, Beijing 100026, China

## Abstract

*Gastrodiae Rhizoma* is a highly valuable traditional herbal medicine commonly used to treat neurological disorders. The present study is designed to determine the antidepressant-like effect of the *Gastrodiae Rhizoma* water extract (GRWE) on a depression model and the potential mechanisms. The chronic unpredictable mild stress (CUMS) rat model was used to induce depression. The sucrose preference test, open field test, forced swimming test, and tail suspension test were performed to assess the depressive-like behaviors, respectively. Hypothalamic–pituitary–adrenal (HPA) function was measured via plasma corticosterone (CORT), adrenocorticotrophic hormone (ACTH), hypothalamic corticotropin-releasing factor (CRF), and glucocorticoid receptor (GR) concentrations. Plasma concentrations of proinflammatory cytokines including interleukin-1*β* (IL-1*β*), interleukin-6 (IL-6), and tumor necrosis factor-*α* (TNF-*α*) were also evaluated. The results showed that GRWE significantly attenuates the behavioral abnormalities in CUMS rats, as shown by elevated sucrose consumption, raised locomotor activity, and reduced immobility duration. Moreover, GRWE treatment reduced CORT, ACTH, CRF, and GR levels and decreased the plasma IL-1*β*, IL-6, and TNF-*α* concentrations. These findings indicate that GRWE improves depressive behaviors in a chronic stress model of rats; its effect may be ascribed to the modulation of the HPA axis activity and inflammatory response.

## 1. Introduction

Depression, a highly debilitating, life-threatening psychiatric disorder, is one of the leading causes of disability worldwide. The World Health Organization predicts depression to be the largest contributor to the disease burden by the year 2030 [1]. The mechanism of depression is complicated, and current antidepressant drugs mainly include monoamine oxidase inhibitors (both reversible and nonreversible), tricyclic and tetracyclic antidepressants, selective serotonin reuptake inhibitors, and serotonin-norepinephrine reuptake inhibitors [2]. However, these antidepressants are not universally effective, and many of them result in undesirable adverse effects, such as sleep disturbance, cognitive impairments, urinary retention, and sexual dysfunction, leading to poor therapeutic compliance [3]. Therefore, there is an urgent need to develop more effective and safer treatments for depression.

Chronic and stressful life events are crucial triggers of depression. In general, stress response-induced hypothalamic–pituitary–adrenal (HPA) axial hyperactivity and activation of the inflammatory response are two important mechanisms related to the onset and development of depression [4]. Growing evidence has shown that the stress response could stimulate depressive behaviors along with hypersecretion of the HPA axis hormones [5]. Moreover, numerous studies support the view that inflammatory disorders are tightly linked with the pathophysiology of depression. Elevated levels of proinflammatory cytokines, especially interleukin-1*β* (IL-1*β*), interleukin-6 (IL-6), and tumor necrosis factor alpha (TNF-*α*), can interact with the neuroendocrine function and influence neuronal function, which has been reported in depression [6]. Thus, antidepressants may alleviate the depressive-like behavior by regulating the HPA activity and inflammatory mediators.

Many functional foods and herbal medicines are gaining attention in efforts to develop alternative therapies to improve the clinical symptoms of depression. *Gastrodiae Rhizoma* (GR) is the dried rhizome of *Gastrodia elata* Blume of the Orchidaceae family. It is a well-known traditional Chinese medicine and a functional food to treat various ailments, including headache, dizziness, convulsion, paralysis, rheumatism, and lumbago, for centuries [7]. GR was reported to possess several biological activities, including antioxidative and free-radical scavenging, neuroprotective, learning improvement, anxiolytic, and antidepressive effects [8]. Recently, GR has been demonstrated to mediate its antidepressive effect via regulating the monoamine oxidase enzyme activity, monoamine concentration and turnover, as well as the hippocampal BDNF-related pathway [9–11]. However, the relevant antidepressive mechanisms of the GR water extract (GRWE) have not been fully elucidated and require further exploration.

The current study was conducted to confirm the antidepressive effects of GRWE in a chronic unpredictable mild stress (CUMS) rat model. Whether the underlying mechanisms contributing to the beneficial effects of GRWE are related to the complex balance between the HPA axis and the inflammatory response is also investigated.

## 2. Materials and Methods

### 2.1. Preparation of GRWE

GR was purchased from the GAP base of Yunnan Zhaotong Yiliang Xiaocaoba (Yunnan, China). GRWE was obtained from the Natural Medicine Chemistry Research Center at the Institute of Medicinal Plant Development (Beijing, China) as previously reported [12]; for details, see Supplementary material. We used a two-step ultrapure water method to extract the GE and obtain the GRWE. The chemical components identified in GRWE were presented in Supplementary Table 1 and Supplementary Figure 1. The final extract was concentrated to a density of 1.1 g/cm^3^.

### 2.2. Animals and Treatments

Male Sprague–Dawley rats (200–220 g) were purchased from Beijing Vital River Laboratory Animal Technology Co., Ltd., Beijing, China. All rats were given free access to sterile food and water and maintained under standard laboratory conditions (22 ± 2°C, 50% ± 10% humidity, with a 12 h photoperiod). All experimental procedures were approved by the Laboratory Animal Ethics Committee of the Institute of Medicinal Plant Development, Peking Union Medical College, and conformed to the “Guide for the Care and Use of Laboratory Animals, 8th edition”.

After acclimation for 10 days, the rats (*n* = 90) weighing 262 ± 24 g were randomized into six groups (15 rats per group). The control group was not stressed and treated by intragastric administration of 2 ml distilled water once a day. In the other five groups, the rats were subjected concurrently to CUMS for 4 weeks. GRWE was dissolved in ultrapure water at a concentration of 5, 10, or 20 g (crude drug)/kg body weight and fluoxetine hydrochloride (FH) at 10 mg/kg for oral administration 1 h prior to daily stress exposure. The doses of GRWE and FH were pre\tested prior to the actual experiment. The overall experimental procedure was shown in Supplementary Figure 2.

### 2.3. Chronic Unpredictable Mild Stress Procedure

The CUMS schedule was performed based on an earlier reported method with minor modifications [13]. The control rats were housed five per cage in a separate room and had no contact with the stressed animals. The stressed rats were singly housed and subjected to the subsequent stressors for 28 days: (1) overnight wet bedding, (2) water deprivation overnight, (3) food deprivation overnight, (4) cage tilting at 45° for 24 h, (5) tail pinching for 1 min; (6) overnight flashing light, (7) inversion of the day/night light cycle, (8) overnight odor, (9) 80 dB white noise for 3 h, (10) 5 min cold swimming at 8°C, (11) overnight crowding, and (12) behavior restriction for 4 h. Two types of stressors were randomly applied each day to produce unpredictable mild stress effects.

### 2.4. Sucrose Preference Test (SPT)

SPT was performed prior to and after CUMS exposure to observe anhedonic-like behaviors [9]. In the first 24 h of the experiment, rats were given two bottles of 1% sucrose solution. Then, both the bottle containing sucrose solution (1%) and bottle of water were given ad libitum in the following 24 h. After 24 h without food and water supply, the rats were allowed to freely drink from two bottles: one filled with sucrose solution (1%) and the other with fresh water. The sucrose solution and fresh water consumption were measured within 1 h. SPT was calculated as sucrose intake/(sucrose intake + water intake) × 100%.

### 2.5. Open Field Test (OFT)

An open field (dimensions: 100 × 100 × 40 cm) was grouped into 25 equal squares. Each animal was gently placed into the center area and given free access to explore the enclosed area for 5 min. All rat movements were automatically monitored using a video tracking system (Shanghai Xinsoft Information Technology Co., Ltd., Shanghai, China). The total movement distance and duration at the center of the field were registered. After each test, the open field surface was thoroughly cleaned with 75% ethanol to remove olfactory cues [9].

### 2.6. Tail Suspension Test (TST)

The TST method was conducted based on the previous literature [14]. Here, rats were individually suspended by the tail above the floor with adhesive tape at about 1 cm from the tail tip for 6 min. The immobility time was measured within the final 4 min. Rats were regarded as immobility only when they remained motionless.

### 2.7. Forced Swimming Test (FST)

In the FST, rats were placed in a cylindrical tank (45 cm in height ×40 cm in diameter) with 30 cm depth water at 24–25°C. The FST is a 2-day procedure including an initial 15 min swimming pretest followed by a 6 min swimming test 24 h later. The immobility time of the last 4 min in the total 6 min period was investigated. A rat was considered immobile when it kept floating in water without struggling except for minor movements essential to hold its head above the water. At the end of each test, the rats were dried in a warmed enclosure and then returned to their cages [14].

### 2.8. Plasma and Hypothalamus Sample Preparation

The blood and hypothalamus were obtained 24 h after the behavioral tests. Briefly, the rats were anesthetized with pentobarbital sodium (40 mg/kg) via intraperitoneal injection (i.p.). When the rats were completely anesthetized, blood samples were collected immediately from the abdominal aorta of each rat in EDTA-coated vacutainers. The rats were then decapitated, and the hypothalamus was carefully isolated on ice and quickly frozen in liquid nitrogen and then kept under −80°C until subsequent assays.

### 2.9. Measurement of Neuroendocrine Hormones and Inflammatory Mediators

Hypothalamus tissues were homogenized in a phosphate-buffered solution, and the supernatants were obtained following centrifugation at 10,000 g for 25 min at 4°C. Plasma levels of CORT, ACTH, IL-1*β*, IL-6, and TNF-*α* and hypothalamic levels of CRF and GR were determined by commercially available ELISA kits (Beijing Expand Biotech Co., Ltd., China). All experiments were conducted based on the kit specifications.

### 2.10. Statistical Analysis

All data are expressed as mean ± standard error of the mean and analyzed by Prism 6 (GraphPad Software). One-way analysis of variance (ANOVA) followed by Tukey's test was used to compare multiple groups, whereas comparisons between two groups were assessed by Student's *t*-test. Significant difference was set as *P* < 0.05.

## 3. Results

### 3.1. Effects of GRWE on SPT

**Figure 1:**
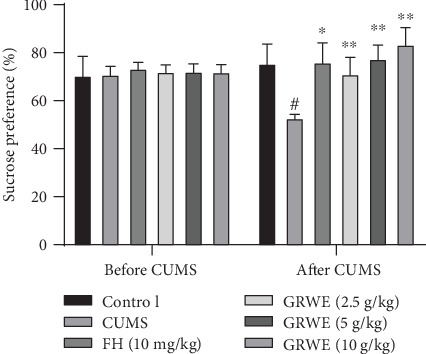
Effects of GRWE on sucrose preference before and after CUMS procedure. Results are expressed as mean ± SEM (*n* = 12). Data were analyzed by *t*-test and one-way ANOVAs, followed by Tukey's post hoc test. ^#^*P* < 0.05 vs. the control group; ^∗^*P* < 0.05 and ^∗∗^*P* < 0.01 vs. the CUMS group. CUMS: chronic unpredictable mild stress; GRWE: *Gastrodiae Rhizoma* water extract; FH: fluoxetine hydrochloride; SEM: standard error of the mean.

The SPT is used to assess the degree of anhedonic-like behavior [15]. As seen in Figure 1, sucrose preference showed no significant differences between groups before CUMS induction. After CUMS procedure for 4 weeks, the sucrose preference of CUMS groups notably reduced in comparison with the control group. All GRWE-treated groups exhibited a remarkable rise in sucrose preference compared with the CUMS group.

### 3.2. Effects of GRWE on the OFT

**Figure 2:**
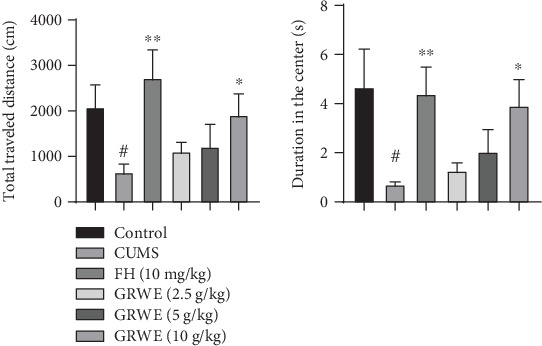
Effects of GRWE on locomotor activity in the open field test after CUMS procedure. Data are presented as mean ± SEM (*n* = 12). Data were analyzed by *t*-test and one-way ANOVAs, followed by Tukey's post hoc test. ^#^*P* < 0.05 vs. the control group; ^∗^*P* < 0.05 and ^∗∗^*P* < 0.01 vs. the CUMS group. CUMS: chronic unpredictable mild stress; GRWE: *Gastrodiae Rhizoma* water extract; FH: fluoxetine hydrochloride; SEM: standard error of the mean.

As shown in Figure 2, in the OFT, both the total traveled distance and duration at the center of the field were lower in the CUMS group when compared with those in the control group. However, the administration of the highest dose of GRWE attenuated these abnormal behaviors.

### 3.3. Effects of GRWE on the TST

**Figure 3:**
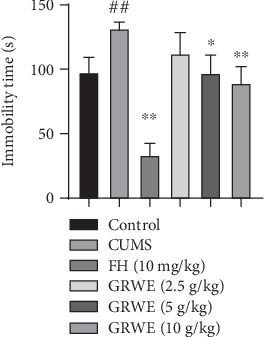
Effects of GRWE on the immobility time in the tail suspension test after CUMS procedure. Results are expressed as mean ± SEM (*n* = 12). Data were analyzed by *t*-test and one-way ANOVAs, followed by Tukey's post hoc test. ^##^*P* < 0.01 vs. the control group; ^∗^*P* < 0.05 and ^∗∗^*P* < 0.01 vs. the CUMS group. CUMS: chronic unpredictable mild stress; GRWE: *Gastrodiae Rhizoma* water extract; FH: fluoxetine hydrochloride; SEM: standard error of the mean.

Figure 3 shows that CUMS resulted in a marked rise in immobility time compared to the control group. GRWE treatment obviously reduced the immobility time of CUMS rats.

### 3.4. Effects of GRWE on the FST

**Figure 4:**
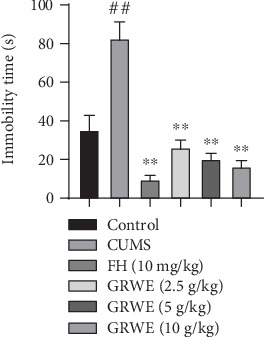
Effects of GRWE on the immobility time in the force swimming test after CUMS procedure. Data are presented as mean ± SEM (*n* = 12). Data were analyzed by *t*-test and one-way ANOVAs, followed by Tukey's post hoc test. ^##^*P* < 0.01 vs. the control group; ^∗∗^*P* < 0.01 vs. the CUMS group. CUMS: chronic unpredictable mild stress; GRWE: *Gastrodiae Rhizoma* water extract; FH: fluoxetine hydrochloride; SEM: standard error of the mean.

In the FST, the duration of immobility in the CUMS group significantly enhanced compared with that of the control group. However, the immobility time was significantly decreased with GRWE treatment under CUMS conditions (Figure 4).

### 3.5. Effects of GRWE on the HPA Axis Activity in the Plasma and Hypothalamus

**Figure 5:**
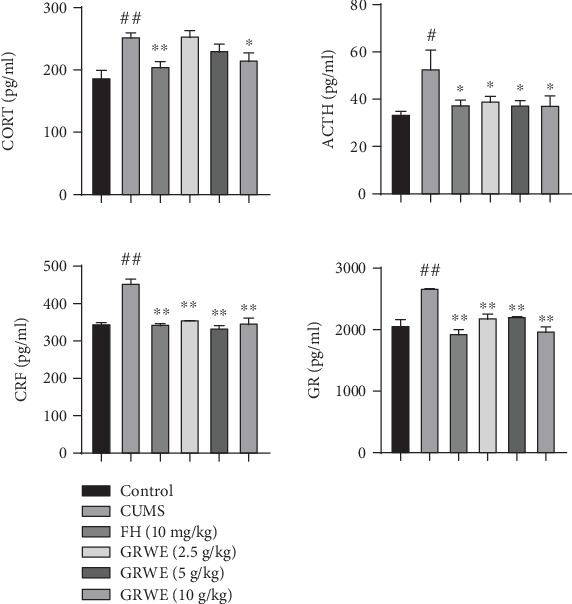
Effects of GRWE on the HPA axis activity in the plasma and hypothalamus in CUMS rats. Data are presented as mean ± SEM (*n* = 12). Data were analyzed by *t*-test and one-way ANOVAs, followed by Tukey's post hoc test. ^#^*P* < 0.05 and ^##^*P* < 0.01 vs. the control group; ^∗^*P* < 0.05 and ^∗∗^*P* < 0.01 vs. the CUMS group. CUMS: chronic unpredictable mild stress; GRWE: *Gastrodiae Rhizoma* water extract; FH: fluoxetine hydrochloride; CORT: corticosterone; ACTH: adrenocorticotrophic hormone; CRF: corticotropin-releasing factor; GR: glucocorticoid receptor; SEM: standard error of the mean.

As shown in Figure 5, the contents of CORT and ACTH significantly increased in the plasma of rats in the CUMS group compared with those in the control group. Treatment with GRWE or FH decreased elevated CORT and ACTH levels. Similarly, hypothalamic CRF and GR levels in CUMS-induced rats were inhibited via GRWE and fluoxetine treatment.

### 3.6. Effects of GRWE on the Production of Proinflammatory Cytokines

**Figure 6:**
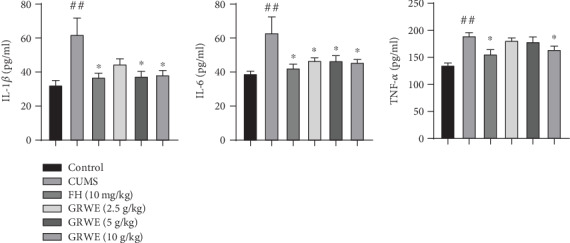
Effects of GRWE on plasma levels of proinflammatory cytokines in CUMS rats. Results are expressed as mean ± SEM (*n* = 12). Data were analyzed by *t*-test and one-way ANOVAs, followed by Tukey's post hoc test. ^##^*P* < 0.01 vs. the control group; ^∗^*P* < 0.05 and ^∗∗^*P* < 0.01 vs. the CUMS group. CUMS: chronic unpredictable mild stress; GRWE: *Gastrodiae Rhizoma* water extract; FH: fluoxetine hydrochloride; IL-1*β*: interleukin-1*β*; IL-6: interleukin-6; TNF-*α*: tumor necrosis factor-*α*; SEM: standard error of the mean.

Figure 6 shows that CUMS significantly elevated the levels of IL-1*β*, IL-6, and TNF-*α* compared with those in the control group. Treatment with GRWE and FH dramatically decreased the plasma IL-1*β*, IL-6, and TNF-*α* concentrations when compared to those in the CUMS group.

## 4. Discussion

The CUMS model, which can simulate the clinical syndrome of depression via stimulating animals with various kinds of stress, is widely used to study the pathophysiology of depression and screen drugs with antidepressive activity [16]. In this research, four parameters were applied to evaluate the animal models of depression: SPT, OFT, FST, and TST. SPT was used to evaluate anhedonia-like behavior, which is a typical clinical manifestation of depressed patients. Locomotor activity in the OFT represents certain aspects of loss of interest, while the immobility time in FST and TST is taken as an index of behavioral despair in depression [17]. In accordance with previous studies, the present results showed successful establishment of the CUMS model for the depressive status by decreases in sucrose intake and locomotor activity and increases in immobility duration. Previous studies show that treatment with FH, a selective serotonin reuptake inhibitor, attenuates CUMS-induced depressive-like behaviors in rats [18], which is in agreement with our results. Notably, GRWE treatment substantially relieved depression-like symptoms in CUMS-induced rats, thus confirming the antidepressant-like effects of the extract.

The HPA axis is a crucial regulatory system regulating the response to stress. Indeed, the hyperactivity of this axis under stress plays a key role in the etiology of depressive disorders [19]. During stress exposure, CRF is released from the hypothalamus and promotes the secretion of ACTH from the pituitary gland to stimulate the release of glucocorticoids (cortisol in humans or CORT in rodents) from the adrenal cortex. Elevated levels of glucocorticoid provide negative feedback to the HPA system by stimulating GR [16]. However, chronic stress disrupts this feedback, which leads to nervous system injury and depression [9, 20]. CUMS has been reported to result in HPA axis hyperactivity, including elevated plasma levels of CORT and ACTH [14]. Consistent with these studies, we observed that plasma concentrations of CORT and ACTH significantly increase in CUMS rats. Previous studies suggest that GRWE treatment improves plasma CORT levels [9]. In addition, our results indicate that GRWE reverses the elevated plasma levels of CORT and ACTH and reduces increased CRH and GR expression in the hypothalamus. These results are consistent with previous studies indicating that GRWE exhibits an inhibitory effect on the HPA axis hyperactivity in CUMS rats.

Large numbers of literature illustrated that peripheral increases in proinflammatory mediators provoked via long-term stress could contribute to depression via immune-mediated signals transmitting from the periphery to the central nervous system [21]. IL-1*β*, IL-6, and TNF-*α* are the most generally demonstrated proinflammatory cytokines in depression [4]. As such, we sought to assess the levels of proinflammatory mediators in the CUMS rat model during GRWE and FH treatment. We showed that CUMS significantly increases the concentrations of IL-1*β*, IL-6, and TNF-*α*, accompanied by behavioral deficits. Our results also found that GRWE treatment could reduce the production of inflammatory cytokines in CUMS rats. This work is the first to report the ability of GRWE to reduce proinflammatory cytokine levels in CUMS-associated depressive disorder. Moreover, it has been documented that the elevated proinflammatory cytokines can promote HPA axis activation; meanwhile, HPA axis activity exerts a consistent regulatory influence on peripheral inflammation, all of which are recognized as potential mechanisms underlying depression [13, 22]. Based on our results, we speculate that GRWE might participate in the crosstalk between inflammatory cytokines and the HPA axis. The reduction of HPA axis overactivation by GRWE may be partly attributed to inhibiting productions of proinflammatory cytokines.

Many researches demonstrated that the active compounds of GRWE, such as gastrodin and 4-hydroxybenzyl alcohol, can cross the brain blood barrier and exhibit neuroprotective effects that may be beneficial in the treatment of neurological disorders [23]. Studies showed that gastrodin could ameliorate the depressive-like behaviors in many preclinical models through regulating neurotransmitters, antioxidation, antiapoptosis, and reducing microglial activation as well as inflammation [24, 25]. Moreover, a clinical trial indicated that gastrodin might be effective for poststroke depression as an adjuvant therapy, but more potent evidence is still necessary [25]. 4-Hydroxybenzyl alcohol exhibited potential antidepressant effects by decreasing monoamine metabolism and modulated cytoskeleton remodeling, which has similar pharmacological effects as gastrodin [23]. These results may be responsible for the antidepressant-like effects of GRWE. However, whether gastrodin or 4-hydroxybenzyl alcohol is truly effective in preventing depression in prospective randomised controlled clinical trials remains to be answered. Further studies on the active components and their synergistically mechanisms of GRWE against depression will be necessary.

## 5. Conclusions

In conclusion, our findings confirm that GRWE could alleviate depression-like behaviors in a rat model of CUMS to an extent similar to that of fluoxetine. This effect maybe partially attributed to the modulation of the HPA axis and inflammatory cytokines. Our results suggest that GRWE has potential use as a beneficial supplement for depression treatment.

## Data Availability

The data used to support the findings of this study are available from the corresponding author upon request.

## Supplementary Materials

Supplementary Materials Supplementary Table 1: the chemical components in GRWE. Supplementary Figure 1: (A) DAD chromatograms of GRWE. (B) Total ion current profile corresponding to the UPLC–ESI-MS analysis of GRWE. Supplementary Figure 2: the outline of design for behavioral tests and the schedule of drug treatment.
