# Supplementary material for: Gastrodiae Rhizoma Water Extract Ameliorates Hypothalamic–Pituitary–Adrenal Axis Hyperactivity and Inflammation Induced by Chronic Unpredictable Mild Stress in Rats
Source: Biomed Res Int. 2020 Jun 10;2020:8374614. doi: 10.1155/2020/8374614 (PMC7305529; doi:10.1155/2020/8374614)
Supplement: Supplementary Materials — Supplementary Table 1: the chemical components in GRWE. Supplementary Figure 1: (A) DAD chromatograms of GRWE. (B) Total ion current profile corresponding to the UPLC–ESI-MS analysis of GRWE. Supplementary Figure 2: the outline of design for behavioral tests and the schedule of drug treatment. [file 8374614.f1.pdf]

Supplementary materials

1. Preparation of GRWE

1.1 Plant material and preparation of Gastrodiae Rhizoma water extract (GRWE)

GR was obtained from the GAP base of Yunnan Zhaotong Yiliang Xiaocaoba (Yunnan, China) in November 2017. A voucher specimen was deposited at the Institute of Medicinal Plant Development (Beijing, China) (No. 201711282). We then used a two-step ultrapure water method to extract the GE and obtain the GRWE. The final extract was concentrated to a density of 1.1 g/cm3.

1.2 UPLC analysis

The samples were separated on an Acquity UPLCTM system (Waters Corp., USA). Chromatographic analysis was conducted using a Waters Acquity-UPLC BEH C18 column (100mm ×2.1 mm i.d., 1.7 μm) at 35℃ and a flow rate of 0.2 mL/min. The mobile phase consisted of 0.1 % aqueous formic acid (A) and acetonitrile (B); the eluting gradient was used as follows: 3–3% B (0–4 min); 3–14% B (4–12 min); 14–40% B (12–16 min); 40–100% B (16–18 min).

1.3 Mass spectrometry

Mass spectrometry detection was performed on a Synapt G2 MS system (Waters Corp., USA) equipped with an ESI source. Two data acquisition modes, or MSE, were selected to investigate precursor ions and product ions. Nitrogen gas was used for nebulization. The detection mode of the flying tube was selected as “V” pattern. The positive ion spectra of the column eluates were recorded at a range of m/z 100–1500. The optimized conditions of the ESI source were listed as follows: capillary voltage, 2.5kV; sampling cone voltage, 40V; extraction cone voltage, 4.0V; ESI source temperature, 120 °C; desolvation temperature, 450 °C; cone gas flow, 50L/h; desolvation gas flow, 800L/h; collision gas flow, 0.5 mL/min. The lock mass compound was leucine encephalin (m/z 554.2615), and the interval scan time was 0.02s. Masslynx4.1(WatersCorp.) software was used to control this instrument.

1.4 Phytochemical analysis of GRWE

GRWE were characterized using UPLC/Q-TOF-MS/MS, in which 6 peaks were characterized in terms of retention times and mass spectra (Supplementary Figure 1). These compounds were then identified by comparing with published data or commercial standards. A complete list summarizing all of the compounds identified in the active fraction is shown in Supplementary Table 1.

Supplementary Table 1: The chemical components in GRWE

Peak	Rt (min)	Chemical name	molecular formula	molecular mass	pseudo-molecular ion peaks	UV
1	3.32	Gastrodin	$C_{13}H_{18}O_7$	286	309 [M + Na]$^+$	220
2	6.90	4-hydroxybenzyl alcohol	$C_7H_8O_2$	124	147 [M + Na]$^+$	220
3	7.04	Benzoic acid	$C_7H_6O_2$	122	145 [M + Na]$^+$	220
4	8.23	Parishin B	$C_{32}H_{40}O_{19}$	728	751 [M + Na]$^+$	280
5	14.10	Parishin C	$C_{32}H_{40}O_{19}$	728	751 [M + Na]$^+$	280
6	14.27	2,4-bis (4-hydroxybenzyl)phenol	$C_{20}H_{18}O_3$	306	329 [M + Na]$^+$	220

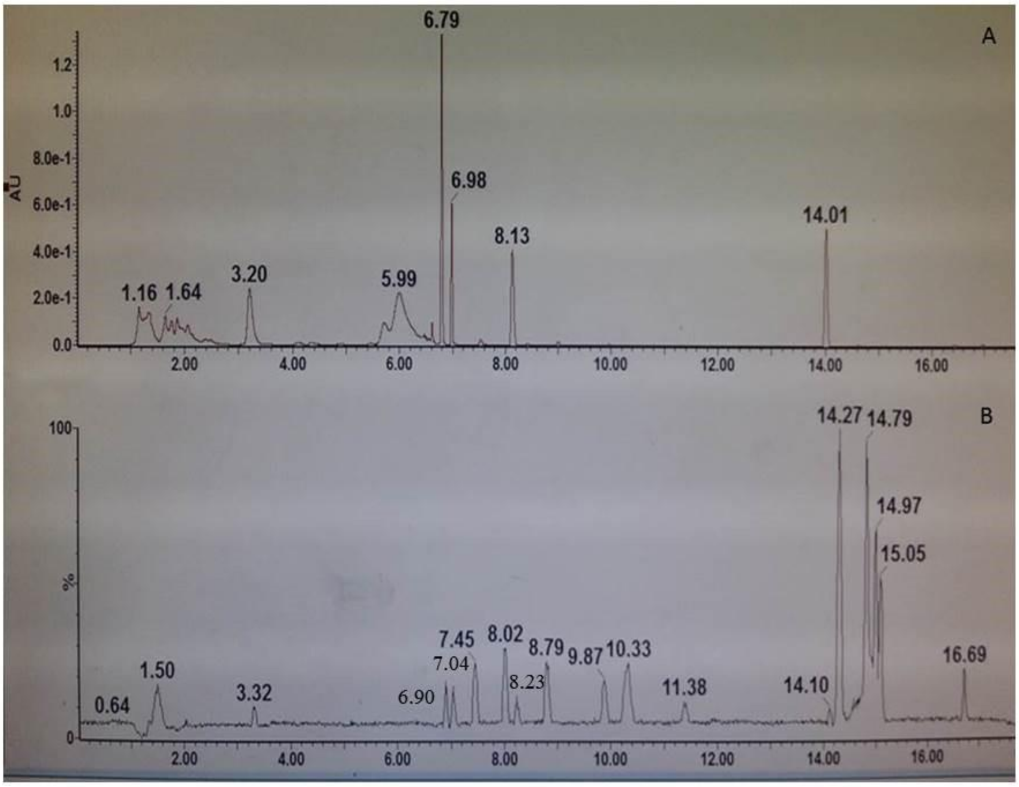

Supplementary Figure 1 (A) DAD chromatograms of GRWE. (B) Total ion current profile corresponding to the UPLC–ESI-MS analysis of GRWE.

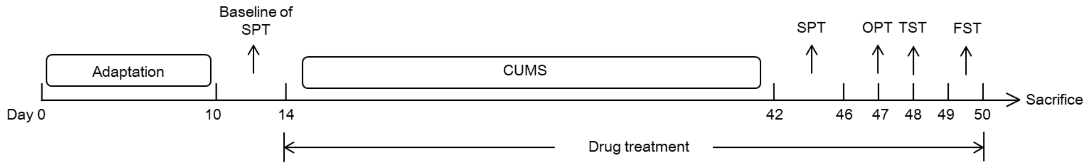

Supplementary Figure 2 The outline of design for behavioral tests and the schedule of drug treatment.
